# Mediterranean diet and risk of multiple sclerosis: A prospective cohort study

**DOI:** 10.1177/13524585251396408

**Published:** 2025-12-15

**Authors:** Sara Ratti, Helén Eke, Anna Cantarutti, Stephanie E Bonn, Hans-Olov Adami, Weimin Ye, Marta Ponzano, Alessandra Grotta, Ylva Trolle Lagerros

**Affiliations:** Department of Statistics and Quantitative Methods, University of Milano-Bicocca, Milan, Italy; Department of Medicine, Huddinge, Karolinska Institutet, Stockholm, Sweden; Department of Statistics and Quantitative Methods, University of Milano-Bicocca, Milan, Italy; Division of Clinical Epidemiology, Department of Medicine, Solna, Karolinska Institutet, Stockholm, Sweden; Department of Medical Epidemiology and Biostatistics, Karolinska Institutet, Stockholm, Sweden; Clinical Effectiveness Group, Institute of Health and Society, University of Oslo, Oslo, Norway; Department of Medical Epidemiology and Biostatistics, Karolinska Institutet, Stockholm, Sweden; Department of Health Sciences, University of Genoa, Genoa, Italy; Department of Public Health Sciences, Stockholm University, Stockholm, Sweden; Centre for Health Equity Studies, Stockholm University/Karolinska Institutet, Stockholm, Sweden; Department of Medicine, Huddinge, Karolinska Institutet, Stockholm, Sweden

**Keywords:** Mediterranean diet, multiple sclerosis, epidemiology, cohort study, nutrition

## Abstract

**Background and Objectives:**

Although adherence to the Mediterranean diet has been reported to be inversely associated with risk of neurodegenerative disease, it is uncertain if this dietary pattern also reduces multiple sclerosis (MS) risk.

**Methods:**

We analyzed data from 41,428 participants in the Swedish National March Cohort. Dietary intake was assessed at baseline in 1997 using a Food Frequency Questionnaire. The Mediterranean Diet Score was analyzed numerically (range: 0–9) and categorically (low, medium, high adherence). Participants were followed using national registries to identify MS cases. Hazard ratios (HRs) with 95% confidence intervals (CIs) were estimated using multivariable Cox proportional hazards models.

**Results:**

During a mean follow-up of 17.6 years, we identified 89 incident MS cases. Each one-point increase in the Mediterranean Diet Score was associated with a 14% lower MS risk (HR = 0.86, 95% CI: 0.75–0.99). When stratified by smoking, we found a 26% lower risk among non-smokers (HR = 0.74, 95% CI: 0.61–0.90), while no risk reduction was seen among smokers. In age-stratified analyses, inverse associations were observed in participants aged ⩽ 45 years (HR = 0.77, 95% CI: 0.64–0.93), but not in those aged >45 years.

**Conclusions:**

Adherence to the Mediterranean diet was inversely associated with MS risk, supporting its potential neuroprotective role.

## Introduction

Multiple sclerosis (MS) is a chronic disease of the central nervous system, characterized by myelin loss, inflammation, and neurodegeneration.^1^ Common symptoms include visual impairment, sensorimotor dysfunction, fatigue, and cognitive deficits.^1^ Onset is usually at ages 20–45 years, and there is a higher incidence in females.^1^ Sweden has one of the highest global prevalence rates of MS (215 per 100,000).^2^ Established and suspected causes of MS include genetic and environmental factors, such as Vitamin D deficiency,^3^ Epstein–Barr virus infection,^4^ smoking,^5^ obesity,^5^ and potentially also diet.^6^ Although the influence of diet remains under examination, the Mediterranean dietary pattern might be protective.^7,8^ This dietary pattern, characterized by a high consumption of fruits, vegetables, olive oil, and omega-3 fatty acids, has previously been associated with a reduced risk of Alzheimer’s and Parkinson’s disease,^9,10^ potentially due to its antioxidant and anti-inflammatory effects. Current evidence, although still limited in scope, suggests that individuals adhering to the Mediterranean diet may have a lower risk of developing MS, pointing to a potentially beneficial role for this dietary pattern.^7,8^ To expand the limited knowledge, we examined the association between Mediterranean diet adherence and MS incidence in a large Swedish prospective cohort.

## Methods

### Study population

The Swedish National March Cohort was established in 1997 during a nationwide fundraising campaign by the Swedish Cancer Society. This 4-day event took place in nearly 3,600 cities and villages across Sweden. Participants were invited to complete a 36-page questionnaire on lifestyle and medical history, and 43,865 responses were collected.^11^ Through the national registration numbers, the cohort was linked to the National Patient Register (inpatient data from 1987, outpatient diagnoses from 2001), the Cause of Death Register, and the Total Population Register (birth, immigration, and emigration data). The study was approved by the ethical review board in Stockholm (1997-205, 2017/796-31) and all participants provided informed consent. To identify MS diagnoses, we used the following International Classification of Diseases (ICD) codes: ICD-7: 345, ICD-8/9: 340, and ICD-10: G35, including both primary and secondary diagnoses.

We excluded participants with incorrect national registration numbers (*n* = 11), those who had died (*n* = 8), were under 18 years of age (*n* = 1,740), or had emigrated (*n* = 43) before start of follow-up. In addition, we excluded female participants with total energy intake outside the range of 500–3,500 kcal/day (*n* = 297), and male participants outside the range of 800–4,000 kcal/day (*n* = 307). These commonly applied cut-offs are used to exclude implausible energy intake.^12^ Finally, we excluded those diagnosed with MS (*n* = 31) prior to the start of follow-up. The final analytical cohort included 41,428 participants.

### Dietary assessment

Participants were followed from 1 October 1997, until the first MS diagnosis, death, emigration, or the end of follow-up on 31 December 2016, whichever occurred first. Baseline dietary intake was assessed using a validated 85-item semi-quantitative Food Frequency Questionnaire.^13^ Participants reported their average consumption for each food item and beverage during the previous year, with standard portion sizes provided. Response options ranged from 0 to 7 times/day, or as follows: 0; 1–3 times/month; 1–2, 3–4, 5–6 times/week; or 1, 2, 3+ times/day, depending on the item. Missing items were treated as no intake, consistent with the procedure used to calculate total energy intake. This approach ensured internal consistency in the dietary data while minimizing potential bias from differential non-response. Energy and nutrient intakes were derived using the Swedish National Food Composition Database.

We calculated average daily consumption, energy-adjusted the nutrients using the residual method, and assessed adherence to the Mediterranean diet using the Mediterranean Diet Score.^14^ The score ranged from 0 to 9, with 1 point assigned for consumption at or above the sex-specific median for each beneficial component (vegetables, fruits and nuts, legumes, grains, fish, and unsaturated-to-saturated fat ratio), and below the median for less favorable components (dairy and meat). For alcohol, 1 point was assigned for intake within specific ranges (5–25 g/day for females, 10–50 g/day for males); otherwise, 0 points were given. We defined categories of adherence as low (0–3 points, reference), medium (4–5 points), and high (6–9 points), in accordance with the original definition of the Mediterranean Diet Score,^14^ thus maintaining consistency with prior studies.

### Statistical analyses

Baseline characteristics are presented as medians (interquartile ranges, IQR) for non-normally distributed continuous variables, means (standard deviations, SD) for normally distributed continuous variables, and frequencies (percentages, %) for categorical variables.

Cox proportional hazards models were applied to estimate hazard ratios (HRs) with 95% confidence intervals (CIs) for the association between Mediterranean diet adherence and MS risk, using age as the underlying timescale. The models were adjusted for several potential confounders: sex (female/male), smoking (non-smoker/smoker), BMI (<25.0/25.0–29.9/≥30.0 kg/m^2^), education level (⩽13/>13 years), leisure time physical activity (tertiles of metabolic energy turnover [MET] hours/week), energy intake (kcal/day), Vitamin D (µg/day), and Vitamin B12 (µg/day). We tested proportional hazards assumptions using Schoenfeld residuals. To examine possible effect modification, we fitted the cross-product interaction term between the dietary score and selected covariates (smoking, education level, and BMI) for each model, and tested statistical significance using the Likelihood Ratio (LR) test. Based on the results, we present stratified analysis by smoking for each model. Given the age-dependent nature of MS incidence, which typically occurs between 20 and 45 years of age,^1^ we additionally fitted models stratified by baseline age category (⩽45/>45 years) to explore potential differences in associations across age groups. We also conducted a sensitivity analysis excluding MS cases diagnosed in the first two years of follow-up (*n* = 2) to avoid reverse causation.

Missing data were as follows: education 1.1%, smoking 2.2%, physical activity 3.9% and BMI 4.5%. Missing values were imputed using regression-based models. Logistic regression was applied for dichotomous variables (education, smoking), and ordinal logistic regression was applied for categorical variables (BMI, physical activity). The predictor variables were specified as follows: for education, predictors included sex, and age; for BMI, predictor were sex, age, total energy intake, and education; for smoking, predictor included sex, age, and education; and for physical activity, predictors were sex, age, education, total energy intake, and BMI. For each missing observation, predicted probabilities were generated, and the most likely category was assigned.

All statistical analyses were performed with Stata version 18.0 (Stata Corporation, Collage Station, TX, USA). *p*-values below 0.05 were considered statistically significant.

## Results

Table 1 shows baseline characteristics according to Mediterranean Diet Score adherence categories. Among the 41,428 participants, the median Mediterranean Diet Score was 4 points (IQR, 3) and the mean age was 51.6 years (SD, 16.0), whereof 32.1% of the population was 45 years or younger. Most participants had a BMI below 25 kg/m^2^ (61.6%), and females accounted for 64.5% of the cohort. Smoking status and dietary intake of Vitamin D and Vitamin B12 were evenly distributed across adherence levels. By contrast, participants with higher adherence to the Mediterranean diet tended to be older, more educated, and reported both a higher energy intake and higher levels of physical activity.

**Table 1.** Baseline characteristics of participants in the Swedish National March Cohort, categorized by adherence to the Mediterranean Diet Score.

		*Adherence to Mediterranean Diet*			
	All	Low (0–3)	Medium (4–5)	High (6–9)	*p*-value
No. of participants	41,428	12,201	18,245	10,982	
Age, y, mean (SD)	51.6 (16.0)	48.3 (17.3)	51.9 (15.8)	55.0 (14.0)	*<* *0.001*
Age, y, %					*<* *0.001*
⩽ 45 years at baseline	32.1	42.1	31.5	22.0	
> 45 years at baseline	67.9	57.9	68.5	78.0	
Energy intake, kcal/d, mean (SD)	2,021 (514)	1,875 (487)	2,030 (518)	2,167 (491)	*<* *0.001*
Vitamin D, µg/d, median (IQR)	3.8 (1.8)	3.6 (1.7)	3.7 (1.8)	3.9 (1.8)	*<* *0.001*
Vitamin B12, µg/d, median (IQR)	6.6 (3.3)	6.6 (3.0)	6.6 (3.4)	6.5 (3.5)	*<* *0.001*
Sex, %					*0.01*
Female	64.5	64.5	63.8	65.5	
Male	35.5	35.5	36.2	34.5	
BMI, kg/m^2^, %					*<* *0.001*
< 25.0	61.6	63.2	60.9	60.8	
25.0-29.9	31.5	29.7	32.0	32.6	
⩾ 30.0	7.0	7.1	7.1	6.6	
Smoking, %					*<* *0.001*
Non-smoker	60.6	62.1	60.6	58.9	
Smoker	39.4	37.9	39.4	41.1	
Total Leisure Physical Activity, tertiles of MET h/week, %					*<* *0.001*
Low, < 9	34.2	38.6	34.0	29.4	
Medium, 9-16.5	32.9	31.3	33.0	34.3	
High, > 16.5	33.0	30.0	32.9	36.3	
Education, y, %					*<* *0.001*
⩽ 13	71.3	74.6	71.2	67.8	
> 13	28.7	25.4	28.8	32.2	

BMI = body mass index, IQR = interquartile range, MET = metabolic energy turnover, SD = standard deviation.

*p*-values were obtained by Kruskal–Wallis test for asymmetrical continuous variables, by ANOVA test for normally distributed continuous variables, and by chi-square test for categorical variables.

During a mean follow-up of 17.6 years, we identified 89 incident cases of MS. As shown in Table 2, a one-point increase in the Mediterranean Diet Score was associated with a 14% lower risk of MS (HR = 0.86, 95% CI: 0.75–0.99) after adjusting for potential confounders. When the score was examined categorically (low, medium, high), results deviated slightly from linearity. We found no reduction in risk for the medium adherence group. Although the high adherence group showed a lower risk compared to the low adherence group, this difference was not statistically significant.

**Table 2.** Multivariable-adjusted HRs with 95% CIs for the association between Mediterranean Diet Score and risk of Multiple Sclerosis in the Swedish National March Cohort.

	Multiple Sclerosis				
Multivariable-adjusted models^a^	No.	IR	HR	95% CI	*p Trend*
Mediterranean Diet Score	89	1.22	0.86	0.75 – 0.99	*0.03*
Mediterranean Diet Score, categorized					*0.07*
Low	24	1.11	1.00	Ref.	
Medium	49	1.52	1.15	0.70 – 1.90	
High	16	0.83	0.53	0.27 – 1.03	

CI = confidence interval, HR = hazard ratio, IR = incidence rates (per 10,000 person-years), No. = number of participants with Multiple Sclerosis, Ref. = reference.

a Adjusted for age (years, model time scale), sex (female/male), smoking (non-smoker/smoker), body mass index (<25.0/25.0–29.9/≥30.0 kg/m^2^), education level (⩽13/>13 years), physical activity (tertiles metabolic energy turnover, MET h/w), energy intake (kcal/d), Vitamin D (µg/d), and Vitamin B12 (µg/d).

We aimed to explore whether the association observed between Mediterranean diet adherence and MS risk differed across key factors known to influence disease onset, specifically smoking and age. Table 3 shows models stratified by smoking status. We found significant interaction terms between Mediterranean diet adherence and smoking for both the numerical (*p* = 0.04) and the categorical (*p* = 0.03) model, with evidence for effect modification by smoking on the multiplicative scale (LR-tests *p* = 0.04 and *p* = 0.04, respectively). Among non-smokers, adherence to the Mediterranean diet was associated with lower MS risk: the numerical model showed a 26% risk reduction per one-point increase in score (HR = 0.74, 95% CI: 0.61–0.90), and categorical analysis indicated that high adherence was linked to a risk reduction of 81% (HR = 0.19, 95% CI: 0.06–0.60) compared to low adherence. In contrast, no statistically significant associations were observed among smokers, with HRs close to unity in both the numerical (HR = 1.00, 95% CI: 0.83–1.22) and the categorical model, with HR = 1.76 (95% CI: 0.78–3.97) for medium versus low adherence and HR = 1.23 (95% CI: 0.48–3.14) for high versus low adherence.

**Table 3.** Subgroup analyses by smoking of the Mediterranean Diet Score on the risk of Multiple Sclerosis.

	Multiple Sclerosis					
Multivariable-adjusted models^a^	No.	IR	HR	95% CI	*p Trend*	*p LR-test*
Mediterranean Diet Score						*0.04*
Non-Smoker	45	1.01	0.74	0.61 – 0.90	*0.002*	
Smoker	44	1.53	1.00	0.83 – 1.22	*0.98*	
Mediterranean Diet Score, categorized						*0.04*
Non-smoker					*0.005*	
Low	16	1.19	1.00	Ref.		
Medium	25	1.28	0.86	0.45 – 1.64		
High	4	0.35	0.19	0.06 – 0.60		
Smoker					*0.77*	
Low	8	0.98	1.00	Ref.		
Medium	24	1.89	1.76	0.78 – 3.97		
High	12	1.52	1.23	0.48 – 3.14		

CI = confidence interval, HR = hazard ratio, IR = incidence rates (per 10,000 person-years), LR = likelihood ratio, No. = number of participants with Multiple Sclerosis, Ref. = reference.

a Adjusted for age (years, model time scale), sex (female/male), body mass index (<25.0/25.0–29.9/≥30.0 kg/m^2^), education level (⩽13/>13 years), physical activity (tertiles metabolic energy turnover, MET h/w), energy intake (kcal/d), Vitamin D (µg/d), and Vitamin B12 (µg/d).

To account for the age-dependent nature of MS incidence, we conducted subgroup analyses by age categories, as shown in Table 4. Among participants aged ⩽ 45 years, higher adherence to the Mediterranean diet was associated with a lower risk of MS. In the numerical model, the HR per one-unit increase in Mediterranean Diet Score was 0.77 (95% CI: 0.64–0.93), corresponding to a 23% reduction in risk. Categorical analysis confirmed this pattern, with a *p*-value for the linear trend of 0.04. While medium adherence was not associated with risk (HR = 0.95, 95% CI: 0.51–1.76), high adherence was linked to a 72% lower risk compared with low adherence (HR = 0.28, 95% CI: 0.09–0.85). In contrast, no associations were evident among participants aged > 45 years, as both the numerical (HR = 1.03, 95% CI: 0.84–1.25) and the categorical model yielded estimates above 1, and the *p*-value for the linear trend was not significant.

**Table 4.** Subgroup analyses by age categories of the Mediterranean Diet Score on the risk of Multiple Sclerosis.

	Multiple Sclerosis				
Multivariable-adjusted models^a^	No.	IR	HR	95% CI	*p Trend*
Mediterranean Diet Score					
⩽ 45 years	47	1.89	0.77	0.64 – 0.93	*0.008*
> 45 years	42	0.87	1.03	0.84 – 1.25	*0.81*
Mediterranean Diet Score, categorized					
⩽ 45 years					*0.04*
Low	18	1.87	1.00	Ref.	
Medium	25	2.33	0.95	0.51 – 1.76	
High	4	0.88	0.28	0.09 – 0.85	
> 45 years					*0.94*
Low	6	0.50	1.00	Ref.	
Medium	24	1.11	1.99	0.80 – 4.91	
High	12	0.81	1.23	0.45 – 3.42	

CI = confidence interval, HR = hazard ratio, IR = incidence rates (per 10,000 person-years), No. = number of participants with Multiple Sclerosis, Ref. = reference.

a Adjusted for age (years, model time scale), sex (female/male), smoking (non-smoker/smoker), body mass index (<25.0/25.0–29.9/≥30.0 kg/m^2^), education level (⩽13/>13 years), physical activity (tertiles metabolic energy turnover, MET h/w), energy intake (kcal/d), Vitamin D (µg/d), and Vitamin B12 (µg/d).

Finally, to account for possible reverse causation, we performed sensitivity analyses excluding MS cases diagnosed within the first 2 years of follow-up (*n* = 2). The results were robust, and the associations between Mediterranean diet adherence and MS risk remained materially unchanged.

## Discussion

In this large prospective cohort study, a higher Mediterranean Diet Score was associated with a substantially lower risk of MS. The association was particularly strong among non-smokers, whereas no association was observed among smokers, suggesting a potential interaction between dietary patterns and smoking. The observed effect modification is consistent with existing evidence indicating that smoking may counteract the beneficial effects of a healthy diet by promoting inflammation and oxidative stress, key factors in MS pathogenesis.^15^ Age-stratified analyses further indicated that the inverse association was restricted to participants aged 45 years or younger, which is consistent with the typical age at MS onset and likely reflects both disease epidemiology and reduced statistical power. These findings underscore the significance of lifestyle factors as potential modifiers of diet-disease relationships. Our results contribute to an emerging, but still heterogeneous body of evidence on the Mediterranean diet and MS. A large Swedish population-based case-control study^7^ reported a 46% reduced MS risk among individuals adhering to a Mediterranean diet, compared with those following a Western-style diet, even after extensive adjustment for lifestyle and environmental confounders. Similarly, a Canadian case-control study^8^ of pediatric MS onset found that each one-point increase in the Mediterranean Diet Score was associated with a 37% lower odds of MS. By contrast, analyses from the UK Biobank^16^ did not find significant associations between Mediterranean diet adherence and MS incidence, although non-significant protective trends were observed. Differences in study design, population age, sample size, and dietary assessment methods likely contributed to these discrepancies, underscoring the challenges of studying a relatively rare disease where statistical power often is limited.

Strengths of our study include its prospective design, the large sample size, and long-term follow-up, which together minimize recall and selection bias and support the temporal relationship between exposure and outcome. The use of a validated Food Frequency Questionnaire enabled detailed assessment of habitual diet, while linkage to national health registers ensured accurate ascertainment of MS cases. In addition, our analysis benefits from adjustment for key potential confounders, such as Vitamin D and B12 intake, which were not always accounted for in previous studies. The availability of detailed data also allowed us to explore potential effect modification by smoking and to stratify by age, providing further insight into subgroups where diet may exert greater influence. Compared with the general Swedish population in 1997, cohort members were less educated, were more frequently overweight or had obesity, and were less likely to smoke.^11^

Nevertheless, our findings are subject to certain limitations. Despite the large cohort and extended follow-up, the rarity of MS led to only 89 identified cases, resulting in wide confidence intervals and limited statistical power, particularly in subgroup analyses. Accordingly, the associations should be interpreted with caution, and our conclusions remain cautious, although the number of cases was in line with a priori expectations. Another important limitation relates to age; the higher mean age of participants at baseline (51.6 years) compared to the usual age of MS onset (20–45 years) likely contributed to the low number of cases and may limit the generalizability of our findings to younger populations at greatest risk of developing MS. In our cohort, the mean age at MS onset was 44.5 years (SD, 13.6), which is higher than the average reported in international epidemiological estimates. In 2008, the global mean age at onset was 29.2 years, with a range of 30–35 years in Sweden.^17^ More recent data from 2020 indicate a gradual upward shift, with an average of 32 years worldwide and 38 years in Sweden.^2^ This trend may partly explain the higher onset age observed in our study, although it probably also reflects the older baseline age of the cohort. In addition, diet was assessed only at baseline, which prevented the examination of dietary changes over time and their potential impact on MS risk. Furthermore, although we adjusted for several important confounders, we lacked detailed clinical information, such as the presence of other autoimmune conditions (e.g. type 1 diabetes, rheumatoid arthritis) or chronic comorbidities, that could account for unmeasured effects. As a result, residual confounding remains possible, particularly given that the prevalence of such conditions increases with age, and our cohort had a relatively old baseline distribution. Finally, unmeasured environmental or genetic factors, such as genetic predisposition, family history of MS, or prior Epstein–Barr virus infection, may have influenced the associations and cannot be ruled out. We also acknowledge that, given the rarity of MS, alternative designs such as case-control studies may offer greater statistical efficiency. However, with a prospective cohort design it is possible to ensure that a dietary information was collected before disease onset, thereby minimizing recall bias and providing a clearer temporal sequence between exposure and outcome.

In conclusion, our findings suggest a potential protective effect of the Mediterranean diet against MS, particularly among younger individuals and non-smokers, but they must be interpreted with caution. Future large-scale longitudinal studies with repeated dietary assessments, ideally with younger and more diverse populations, and careful adjustment for genetic and environmental risk factors, are needed to confirm and expand upon these findings.
